# Foot problems in patients with systemic lupus erythematosus; under-reported and under-treated?

**DOI:** 10.1186/1757-1146-8-S2-O31

**Published:** 2015-09-22

**Authors:** S Otter, S Kumar, P Gow, N Dalbeth, KA Davies, S Panthakalam, K Rome

**Affiliations:** 1 Auckland University of Technology, Auckland, New Zealand; 2 Middlemore Hospital, Papatoetoe 2025, New Zealand; 3 The University of Auckland, Auckland, 1010, New Zealand; 4 Brighton & Sussex Medical School, Brighton, BN1 9PX, UK; 5 Eastbourne District General Hospital, Eastbourne, BN21 2UD, UK

**Keywords:** systemic lupus erythematosus, foot, podiatry

## Background

Foot pathology is common in inflammatory arthritis and the role of the podiatrist in the multidisciplinary care team is well established. However, in systemic lupus erythematosus; (SLE) the need for foot health services and service provision for foot disease is unknown. We set out to determine the perceived need and uptake of foot care services.

## Methods

A 40-item self-administered postal questionnaire was posted to patients with SLE attending adult rheumatology clinics at Auckland and Counties Manukau District Health Boards, Auckland, New Zealand. The questionnaire enquired about the occurrence of foot symptoms and their frequency of assessment, the availability of podiatric services and the usefulness of interventions.

## Results

In total, 107 patients responded with 79% reporting foot pain caused by their SLE. Half (51%) of the patients had discussed foot pain with their general practitioner or rheumatologist, and a third (33%) had difficulty with basic foot care. Respondents reported there was no significant difference in the frequency with which their hands and feet were examined. However, only 33% had been seen by a podiatrist. Insoles had only been prescribed to a quarter of respondents (25%) but only half of those receiving insoles were continuing to wear them and merely two respondents indicated their foot symptoms had been resolved by their insoles. None of the subjects reported that they had been provided with specialist footwear.

## Conclusion

These data suggest that foot problems are common and under-reported in patients with SLE. Health care professionals need to consider a comprehensive foot care plan as part of the holistic management of people with SLE.

